# ACL Surgery Necessity in Non-Acute Patients (ACL SNNAP): a statistical analysis plan for a randomised controlled trial

**DOI:** 10.1186/s13063-022-06309-6

**Published:** 2022-05-12

**Authors:** Jamie R. Stokes, David J. Beard, Loretta Davies, Beverly A. Shirkey, Andrew Price, Jonathan A. Cook, The ACL SNNAP Study Group, Karen Barker, Andrew Carr, Jonathan Cook, Loretta Davies, Fares Haddad, William Jackson, Sallie Lamb, Jose Leal, Paul Monk, Sean O’Leary, Andrew Price, Chris Wilson

**Affiliations:** 1 Oxford Clinical Trials Research Unit, Centre for Statistics in Medicine, Nuffield Department of Orthopaedics, Rheumatology, and Musculoskeletal Sciences, University of Oxford, Oxford, OX3 7LD UK; 2 Surgical Interventional Trials Unit, Nuffield Department Orthopaedics, Rheumatology and Musculoskeletal Sciences, University of Oxford, Oxford, UK; 3 School of Social and Community Medicine, University of Bristol, Canynge Hall, Bristol, UK; 4 Nuffield Orthopaedic Centre, Nuffield Department of Orthopaedics, Rheumatology, and Musculoskeletal Sciences, University of Oxford, Oxford, OX3 7LD UK

**Keywords:** Anterior cruciate ligament, ACL, Randomised controlled trial, Statistic, Analysis plan, Reconstruction, Rehabilitation

## Abstract

**Background:**

Rupture of the anterior cruciate ligament (ACL) is a common injury, primarily affecting young, active individuals. Despite surgical intervention being the more common treatment for patients suffering ACL ruptures, current management is based on limited and generally low-quality evidence. We describe a statistical analysis plan (SAP) for the ACL SNNAP randomised controlled trial, which aims to investigate the necessity of surgical management in patients with ACL injuries.

**Methods/design:**

ACL SNNAP is a pragmatic, multi-centre, superiority, parallel-group randomised controlled trial in participants with a symptomatic non-acute ACL deficient knee. Participants are allocated in a 1:1 ratio to either non-surgical management (rehabilitation) or surgical management (reconstruction) with the aim of assessing the efficacy and cost-effectiveness. The primary outcome of the study is the Knee Injury and Osteoarthritis Outcome Score (KOOS4) at 18 months post-randomisation. The KOOS4 score at 18 months will be evaluated using a linear regression model adjusting for recruitment centre and baseline KOOS4 scores, allowing for intra-centre correlation. A secondary analysis of the primary outcome will be carried out using an area under the curve (AUC) approach using treatment estimates obtained from a mixed model using baseline, 6 months, 12 months, and 18 months post-randomisation outcome data. Secondary outcomes will be measured at 18 months and will include return to activity/level of sport participation, intervention-related complications, the EQ-5D-5L questionnaire, all 5 individual subscales of the KOOS questionnaire, the ACL-QOL score, expectations of return to activity and cost-effectiveness of the interventions. Missing primary outcome data will be investigated through a sensitivity analysis. Full details of the planned methods for the statistical analysis of clinical outcomes are presented in this paper. The study protocol for the ACL SNNAP trial has been published previously.

**Discussion:**

The methods of analysis for the ACL SNNAP trial have been described here to minimise the risk of data-driven results and reporting bias. Any deviations from the analysis methods described in this paper will be described in full and justified in the publications of the trial results.

**Trial registration:**

ISRCTN ISRCTN10110685. Registered on 16 November 2016

## Background

Anterior cruciate ligament (ACL) rupture is a common injury which mainly affects young, active individuals with an estimated 200,000 injuries occurring annually in the USA [1]. ACL injury can lead to recurrent knee instability (giving way) [2], as well as poor quality of life, decreased activity [3] and increased risk of secondary osteoarthritis of the knee [4]. Whilst some patients are able to function well without the ACL after some formal rehabilitation [5], other patients with episodes of knee instability are thought to require surgery to stabilise the knee.

In England, it is conservatively estimated that around 15,000 primary ACL reconstruction surgeries are performed every year [6], with around 80% of non-acute patients now listed directly for surgery in the NHS. However, it is possible that this estimate is closer to 50,000 per year based on the size of the UK population and incidence data taken from the Swedish ACL registry [7]. Despite these figures, a 2009 Cochrane review into whether surgical or non-surgical management was superior for ACL injury concluded that no high-quality evidence exists on which to base practice [8], with an update to the review in 2016 drawing similar conclusions [9]. In contrast to Frobell et al.’s findings [10], a recent paper [11] reported that for a group of acute ACL-injured patients, those who underwent early surgical reconstruction had improved symptoms/knee function and activity at 2 years compared to those who first had rehabilitation (followed by optional elective surgical reconstruction). However, similarly to Frobell et al.’s study, a significant proportion of patients (50%) who underwent rehabilitation did not require ACL reconstruction.

The ACL SNNAP randomised controlled trial aims to address the gap in evidence base regarding the clinical and cost-effectiveness of the surgical and non-surgical management approaches for patients suffering ACL injuries and inform standards of care.

This paper describes the methods set out in the trial’s statistical analysis plan and has been prepared in accordance with published guidance on the contents of statistical analysis plans [12]. The publication of this paper will provide transparency on the planned analysis strategies relating to the clinical outcomes chosen to compare the approaches to ACL injury management. This will ensure the risk of reporting bias and data-driven results is minimised. The ACL SNNAP trial is registered in the International Standard Randomised Controlled Trials (ISRCTN) database (registration number ISRCTN10110685).

## Methods and design

### Trial design

ACL SNNAP is a pragmatic multi-centre randomised controlled trial with two-arm parallel groups and a 1:1 allocation ratio to compare non-surgical management (rehabilitation) and surgical management (reconstruction) options for patients with a symptomatic non-acute ACL-deficient knee. An internal pilot will be conducted with clear progression criteria regarding recruitment. The target recruitment was 320 participants. Recruitment is now closed, and study follow-up is currently underway. Randomisation was performed using a web-based automated system. The allocation was generated using permuted block randomisation with varying block sizes stratified by baseline KOOS score (< 30 or ≥ 30) and recruitment site. Other than the allocated intervention, both groups are being followed up in the same way to exclude bias. Follow-up for study purposes is by patient self-reported questionnaire completed using an electronic data capture collection system (a postal option will also be available). The questionnaire is completed by participants at baseline and at 6, 12 and 18 months. Full details of the trial design and procedures have been published previously in the trial protocol [13].

### Current status of trial

Recruitment for ACL SNNAP began on 1 February 2017 and concluded on 12 April 2020, recruiting 316 participants. Follow-up will be completed for all participants by January 2022. The trial SAP was finalised and submitted for publication prior to completion of the follow-up of the final participants and before the beginning of the final analysis.

### Objectives

The main objective of ACL SNNAP is to assess whether a strategy of non-surgical management (with the option for later ACL reconstruction only if required) is more clinically effective and cost-effective than surgical management in patients with non-acute anterior cruciate ligament deficiency (ACLD). This will be determined using the KOOS4 score as the comparative measure. Secondary clinical objectives will include the differences in return to activity/level of sport, generic quality of life, knee-specific patient-reported outcomes, intervention-related complications, expectations of return to activity and participant satisfaction with the treatment. Cost-effectiveness is also a secondary objective and will be assessed via a within-trial health economic analysis.

The format and design of the trial are based on an assessment of the longitudinal management (treatment strategy) of non-acute ACL injury, rather than a specific direct parallel comparison of two entirely independent treatment interventions. In one of the arms (non-surgical), there are permitted and expected adjustments to treatment content (in the light of unsuccessful first-line approaches) which include the provision of ACL reconstructive surgery (the same intervention as that provided in the comparative study arm at the outset). This, and the complex nature of each intervention, will provide some analytic and interpretation challenges.

### Outcomes

#### Primary outcome

The primary outcome of the study is the Knee Injury and Osteoarthritis Outcome Score (KOOS4) at 18 months post-randomisation. This outcome measure is derived from 4 of 5 subscales: pain, symptoms, difficulty in sports and recreational activities, knee-related quality of life [14, 15] with scores ranging from 0 to 100 and a higher score indicating better health. KOOS is a validated patient-reported outcome used in ACL research (including a recent RCT of acute ACL patients [10] and large scale databases, i.e. National Ligament Registry [16, 17]). The KOOS4 is sensitive and specific for detecting functional deficits due to knee instability.

Patients will complete the KOOS4 scores at baseline (i.e. prior to treatment) and at 6 months, 12 months, and 18 months post-randomisation. The 18-month KOOS4 score will be analysed in a model including the baseline KOOS4 score.

#### Secondary outcomes

The principal time point of interest is 18 months post-randomisation and the level at that point or events occurring up to that point for the respective outcomes.

##### Return to activity/level of sport participation

Activity level will be assessed using the Tegner Scale [18], graded from 1 (low activity levels) to 10 (professional level). In addition, the Tegner has been modified as follows: three columns with the headings of (1) activity level before your injury (select one level only), (2) current level of activity (today) (select one level only) and (3) level you expect to return to (select one level only) were added to the baseline form. At 18 months, the Tegner contains one answer column as follows: current level of activity (today) (select one level only).

##### Intervention-related complications

Any complications associated with undergoing ACL deficiency treatment will be recorded. For the surgery group, these will include re-admission, delayed hospital discharge, infection, poor range of movement (stiffness), excess bleeding, continued swelling, episodes of giving way and continued feeling of instability. For the non-surgical group, complications will include continued swelling and episodes of giving way.

##### Generic quality of life

The EuroQol EQ-5D is a validated, generic, self-reported outcome measure covering 5 health domains and used to facilitate the calculation of quality-adjusted life years (QALYs) in health economic evaluations. The original EQ-5D Questionnaire contained 3 response options within each of 5 health domains (mobility, self-care, usual activities, pain/discomfort and anxiety/depression) [19]. More recently, the EQ-5D-5L has been developed to overcome problems with ceiling effects and to improve sensitivity [20]. The 5L version consists of the same five domains as the original but with 5 response options.

##### KOOS subscales

All 5 subscales of the KOOS [14] will be included as separate outcomes (the fifth scale being activities of daily living).

##### Anterior Cruciate Ligament Quality of Life Score

Anterior Cruciate Ligament Quality of Life Score (ACL-QOL) [21] is a validated 32-item, knee-specific measure for chronic ACL deficiency, divided into 5 subscales which include symptoms and physical complaints, work-related concerns, physical activity and sports participation, lifestyle issues and social and emotional concerns. The overall score is calculated (0–100) with higher scores indicating a better outcome.

##### Expectations of return to activity and confidence in relation to the knee

Patients will be questioned on their expected outcome in relation to return to activity, and how confident they feel about doing so, considering any limitation related to their injured knee. This will be assessed using the Tegner Activity Score [18].

##### Patient satisfaction

A simple Likert scale will be used to assess patient satisfaction with the outcome of their treatment.

##### Cost-effectiveness

Detailed resource use data on initial treatments received (surgical reconstruction or rehabilitation) and on subsequent healthcare contacts including re-operations (surgery arm), subsequent surgical reconstructions (rehabilitation arm), surgery-related complications, further rehabilitation and primary care and other secondary care contacts out to 18 months post-randomisation will be assessed. In addition, data will be collected on the ability to work (e.g. sickness absences/return to work number of days off work and subjective working ability). This outcome will not be explored further in this SAP.

### Sample size

The minimal clinically important change (MIC) for the KOOS score has been suggested to be 8–10 points [14]. Estimates of the minimal detectable change (MDC) for the two KOOS subscales most relevant for ACLD vary between 5 and 12 points (symptoms 5–9 and Sport/Rec 6–12) [14]. Conservatively, a target difference of 8 points and a standard deviation of 19 (the highest value observed in a trial of acute patients at baseline amongst the KOOS subscales) were assumed. Given these assumptions, 120 participants per group were required (240 in total) to achieve 90% power at a 2-sided 5% significance level in the absence of any clustering of the outcome. However, in order to ensure sufficient power, clustering (clsampsi Stata command [22]) has been allowed for by conservatively assuming an intracluster correlation (ICC) of 0.06 [23] and cluster size *n*, mean (SD) of 26, 5 (12) and 43, 3 (5) for ACL reconstruction and rehabilitation groups, respectively. This leads to the larger number of 130 participants per group (260) for which just over 80% power is achieved. Given the conservative nature of the assumed values and the anticipated gain in precision from adjusting for the baseline scores and other randomisation factors, actual power was thought to be likely higher even in the presence of clustering. In order to additionally allow for just over 15% missing data (response in a similar trial [10]), 320 participants were needed.

### Statistical analysis

#### General analysis principles

All principal analyses will be based on the intention-to-treat principle (“as randomised”), analysing participants in the groups to which they are randomised irrespective of compliance with treatment allocation. The main analyses will be carried out once the 18-month time point has been reached by the last participant. Any analyses carried out not specified here will be noted as post hoc in the statistical report and any corresponding publication. Analyses will be carried out on a complete case basis, with no imputation for missing data unless specified otherwise.

A single set of trial result analyses is planned once data collection is completed for the study. A single interim analysis was carried out after 100 participants were recruited to estimate the magnitude of clustering for the 6-month KOOS4 outcome data, and a decision was taken not to increase the target sample size on the basis of this result. This decision was made based upon the recommendation of the Data Monitoring Committee which reviewed the interim data.

Two per-protocol analyses are planned for the primary outcome, excluding patients (in both groups) who did not fulfil the minimal protocol criteria. The patients to be excluded from these analyses are described in the “Analysis of primary outcomes” section.

Analyses will be carried out at a 5% level of significance, with 95% confidence intervals and the corresponding *p*-values reported.

#### Descriptive analyses

**Fig. 1:**
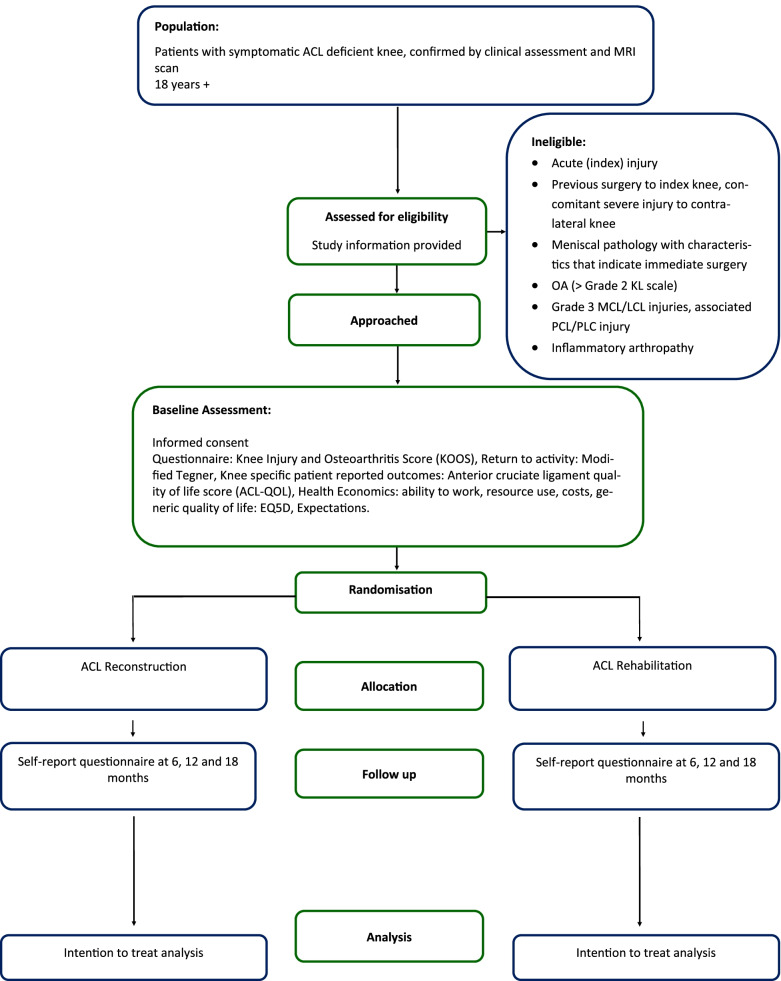
Participants’ flow diagram

A Consolidated Standards of Reporting Trial (CONSORT) diagram will be used to summarise the participants’ flow through the ACL SNNAP trial [24] (Fig. 1). The screening process will be detailed in full, including reasons for ineligibility, exclusion from the final analysis, loss to follow-up and the number of participants who withdrew from the trial before each of the follow-up time points. Numbers (with percentages) for binary and categorical variables and means (and standard deviations) or medians (with lower and upper quartiles) for continuous variables will be presented; there will be no tests of statistical significance nor confidence intervals for the differences between randomised groups on any baseline variable including baseline outcome scores. The baseline characteristics reported will be the randomisation stratification factors (high/low KOOS4 score at randomisation and recruiting centre), sex, age, knee side, time since injury and KOOS4 score as a continuous variable.

#### Loss to follow-up, withdrawals and missing data

Numbers and percentages of withdrawals from each treatment arm will be reported relative to the follow-up time points, together with reasons for withdrawal where available. Differences in withdrawals between the treatment groups will be informally compared, with reasons for any potential differences explored. Surgery after 3 months of rehabilitation is not considered withdrawal from the rehabilitation arm.

Numbers and percentages of data missing for each of the primary and secondary outcomes will be reported. The principal analyses will be performed on an intention-to-treat (ITT) basis, according to randomisation with item-level missing data for the primary outcome dealt with according to the KOOS scoring manual [25] for the primary outcome analysis. However, participant-level missing data will not be imputed in the principal analyses. The impact of missing data at the participant level will be explored via sensitivity analyses for the primary outcome.

#### Compliance

The ACL SNNAP trial involves numerous potential treatment pathways due to the complex nature of the interventions. The potential pathway profiles are described below.

1. Intention to treat profiles
  - S: all patients allocated to surgery (*surgical reconstruction*)
  - R: all patients allocated to rehabilitation (*initial non-surgical management*)

In addition to the principal ITT analysis-based summaries of the groups, descriptive summaries of patients who completed treatment (within treatment protocol) but with different treatment profiles are planned. These profiles are as follows:

2. Complete pathway profiles (intervention as intended)

- SCom: allocated surgical reconstruction, had surgery, completed post-operative rehabilitation
- RCom: allocated rehabilitation, completed rehabilitation, no reconstruction
- RCom S: allocated rehabilitation, completed rehabilitation but underwent surgery, completed post-op rehabilitation

As previously stated, having surgery in the rehabilitation arm (for some patients) is expected and part of the protocol. Some participants in the rehabilitation arm will require surgery, but those participants that do receive surgery having been allocated to non-surgery in the first instance will be analysed as randomised in the principal analysis of the primary outcome.

Some patients do not complete their allocated/intended treatment. For the per-protocol analyses, a further set of patient profiles will be categorised according to any deviation from the allocated pathway (listed 6–12 below as incomplete pathway profiles). Note that “reconstruction or surgery” refers to a decision to list for surgical reconstruction and not necessarily the point in time of the surgical procedure.

3. Incomplete pathway profiles—allocated surgical reconstruction (group S)

- SX: did not have surgery (never had ACLR)
- SX R: did not have surgery, underwent rehabilitation
- SX AS: did not have surgery, still awaiting surgical reconstruction (at 18 months)
- SCom IR: completed surgery but insufficient follow-up time/post-operative rehabilitation (as surgery was delayed)

4. Incomplete pathway profiles—allocated rehabilitation (group R)

- RX: did not start rehabilitation (never had any rehab)
- RI: started rehabilitation but insufficient rehabilitation or unknown rehab completion
- RCom S IR: completed rehabilitation but underwent surgery, insufficient post-operative rehabilitation

#### Analysis of primary outcomes

The principal analysis of the primary outcome measure (KOOS4 score) will be compared using a linear regression model including the treatment arm, with adjustment for the stratification by site (using cluster-robust standard errors) and KOOS4 baseline score. The model will include the KOOS4 score at baseline as a continuous variable and use the cluster option [26] to adjust for stratification by site.

Clustering will be quantified as the intraclass correlation coefficient (ICC) with associated 95% confidence interval calculated using a bootstrapping approach.

A table for the primary outcome will be presented, both with adjusted and unadjusted estimates.

Two per-protocol (PP) analyses are planned, excluding patients who did not fulfil the minimum protocol criteria.

- Conservative PP analysis: excludes all patients that did not fulfil the requirements of the trial for each intervention stated in the protocol (i.e. all the deviations listed above (6–12) in the incomplete pathway profiles)
- Pragmatic PP analysis: replicates the conservative PP analysis above but does *not exclude* patients that had insufficient physiotherapy or did not complete the physio treatment (as can occur as per normal clinical experience)

A secondary analysis of the primary outcome will be performed on the ITT population using an area under the curve (AUC) approach. The treatment estimates obtained from a mixed model at each time point (baseline, 6 months, 12 months, 18 months) will be used to calculate the AUC. The model will include repeated measures of the KOOS4 score (level 1), nested within participants (level 2) and adjusted for the recruitment site as a random effect (level 3). A treatment by time interaction will be included in the model.

Sensitivity analyses will explore the impact of missing data on the main primary outcome ITT analysis. The Stata package rctmiss [27] will be used to show graphically the difference in treatment effect for each arm if different means are assumed for the missing data. A pattern-mixture model will be used to extend the linear regression model for the primary outcome.

A second sensitivity analysis will consider the three responder criteria proposed by Roos et al. as an alternative measure of assessing the KOOS score [28]. The three measures MIC (minimal important change—improvement in the change of KOOS4 > 9), PASS (patient acceptable symptom state—KOOS4 score ≥ 79) and TF (treatment failure—KOOS4 score ≤ 42) will be tabulated by treatment arm, but no formal statistical comparison will be performed.

#### Analysis of secondary outcomes

Unless otherwise specified, the secondary outcomes will be analysed using generalised linear regression models with adjustment for randomisation and baseline variables as described in the analysis of the primary outcome.

#### Return to activity/level of sport participation—Modified Tegner Score

A table of the Modified Tegner Scores will be presented for each time point, one row for each 10 levels and one row for missing data.

The number of participants who did not reach the expected Modified Tegner Score stated at baseline will be tallied, and the table of the expected recovery at baseline vs the 18-month Modified Tegner will be presented.

The difference in Tegner Activity Score between the treatment groups will be assessed at 18 months using a Mann-Whitney *U* test. Return to pre-injury activity level or better will also be assessed using the level each participant stated they were before their injury and the level they reach by 18 months. Confidence intervals for the proportions who returned to their pre-injury activity level will be calculated and reported.

#### Intervention related complications

A table of complications from occurring from surgery to discharge will be reported by treatment arm, with numbers reported for each complication. Details of any further complications will be summarised.

#### Generic quality of life

The VAS and index score will be tabulated reporting the number of observations, means and standard deviations at baseline and 18 months, split by treatment arm. The outcome will be the EQ-5D-5L Index Score and will be analysed using linear regression. The results of the complete case analysis reporting the mean difference and 95% CI will be reported in the table of the analysis results. The model will also be adjusted for the baseline EQ-5D-5L scores, recruitment site (by clustering) and baseline KOOS4 scores.

#### KOOS subscales

The subscales of the KOOS score (pain, symptoms, function in activities of daily living, function in sports and recreation, knee-related quality of life) will be analysed separately in the same way as the primary outcome. Linear regression models adjusting for recruitment site (by clustering) and baseline KOOS scores will be fit to each subscale, with mean differences reported together with 95% CIs and *p*-values.

#### Anterior Cruciate Ligament Quality of Life Score

The ACL-QoL outcome measure is a 32-item questionnaire, with each item being a score between 0 and 100. An overall score is obtained by taking the mean value of these 32 scores, weighting each item equally. Each patient will therefore obtain an overall score between 0 and 100.

ACL-QoL scores will be analysed at the 18-month time point using linear regression, adjusting for recruitment site, baseline KOOS4 scores and baseline ACL-QoL scores. The mean differences will be reported together with 95% CIs and the corresponding *p*-value.

#### Patient satisfaction

Patient satisfaction will be assessed in two ways. Patients will be asked about the nature of their problems at the 18-month time point compared to before their treatment and also if they would still choose to have the same treatment if they were able to go back in time.

For the first question, patients are asked if their knee is much better, a little better, about the same, a little worse or much worse at 18 months compared to before they underwent treatment. This question will be dichotomised for analysis into either “better than before” or “not better than before”. Numbers and proportions of those in each category will be reported by treatment arm, along with confidence intervals for proportions.

The second question asks patients if they would still choose the treatment they received if they could go back in time, with the options “yes”, “no” and “unsure”. The results of this outcome will be assessed descriptively, with numbers and percentages reported by treatment arm.

#### Safety

Any serious adverse events (SAEs) will be reported and described in the text of the report, noting the randomised arm and actual treatment received. If more than 5 SAEs occur, these will be tabulated in the final report by treatment arm.

#### Subgroup analyses

Exploratory subgroup analyses will explore the possible treatment effect modification of clinically important baseline factors (age, gender, high versus moderate or light physical activity as measured by the Modified Tegner Score and KOOS4 overall score) using treatment by factor interactions. The statistical significance level will remain at the 2-sided 5% level, and results will be interpreted cautiously and labelled as “exploratory”.

#### Supplementary/additional analyses and outcomes

The planned supplementary analyses are a complier average causal effect (CACE) analysis, a COVID-19 exploratory analysis and an alternative time window for the primary outcome analysis.

##### Complier Average Causal Effect (CACE)

The study was designed to test the benefit of a treatment policy, to determine the effectiveness of the pathways of rehabilitation first, or surgery first. However, the study findings may be criticised due to the presence of non-compliance. To strengthen the support of the treatment policy, an estimation of the efficacy will be made, with caveat that this study was designed to estimate the effectiveness, not efficacy. The impact of non-compliance will be explored via a CACE analysis. Compliance will be defined as having had surgery at any time (e.g. profiles 3, 5, 9, 12 defined in the “Compliance” section).

##### COVID-19

The COVID-19 pandemic has significantly disrupted all medical research, including the ACL-SNNAP trial. To determine the extent of the effect the pandemic had on SNNAP, the number of patients affected by the pandemic (after the first UK nationwide lockdown on 23 March 2020) will be explored and reported descriptively.

##### Primary outcome analysis with 12–18-month window

In a supplementary analysis, the primary outcome analysis on the ITT population will be repeated using KOOS4 scores collected at 12 months for participants for whom the 18-month outcome data is not available and where sufficient time has passed for the participant to recover from treatment.

#### Statistical packages

All analyses will be carried out using Stata. The relevant package and version number used for analysis will be recorded and reported.

## Discussion

ACL SNNAP will provide valuable data on the clinical and cost-effectiveness of two management strategies (surgical and non-surgical) for non-acute injuries to the ACL. This paper provides details of the statistical analysis that will be carried out once data collection for the trial is complete, with the aim of reducing the risk of data-driven results and reporting bias. A clear principal analysis for the primary outcome and for the secondary outcome has been set up. However, careful interpretation in light of the delivery of the treatments will clearly be needed. As a trial comparing two management strategies, which can be delivered in various ways, a single analysis cannot fully capture all of the anticipated complexity. This complexity has been further heightened by the impact of the COVID-19 pandemic on the delivery of treatments and the impact on the follow-up. Accordingly, the treatment pathway of individual patients will be clearly described and a number of further analyses which seek to explore the impact of treatment delivery and allocation compliance on the trial results.

## Trial status

Recruitment to the ACL SNNAP trial closed on 12 April 2020. A total of 316 patients were recruited in total from 29 sites. Follow-up is expected to finish in January 2022, with both the statistical and health economic analyses to be conducted once this is complete. Any changes or deviations from the planned analyses set out here will be documented and justified fully in the final report.

## Data Availability

Not applicable.

## Abbreviations

ACL: Anterior cruciate ligament
ACL QOL: Anterior Cruciate Ligament Quality of Life Score
ACL SNNAP: ACL Surgery Necessity in Non-Acute Patients
ACLD: Anterior cruciate ligament deficiency
AUC: Area under the curve
CACE: Complier average causal effect
CI: Confidence interval
CONSORT: Consolidated Standards of Reporting Trial
ICC: Intraclass correlation coefficient
ITT: Intention-to-treat
KOOS: Knee Injury and Osteoarthritis Outcome Score
MDC: Minimum detectable change
MIC: Minimal important change
PASS: Patient Acceptable Symptom State
PP: Per-protocol
QALY: Quality-adjusted life year
SAE: Serious adverse event
TF: Treatment failure
VAS: Visual analogue scale
